# Discovering new mTOR inhibitors for cancer treatment through virtual screening methods and *in vitro* assays

**DOI:** 10.1038/srep18987

**Published:** 2016-01-06

**Authors:** Ling Wang, Lei Chen, Miao Yu, Li-Hui Xu, Bao Cheng, Yong-Sheng Lin, Qiong Gu, Xian-Hui He, Jun Xu

**Affiliations:** 1 Research Center for Drug Discovery & Institute of Human Virology, School of Pharmaceutical Sciences, Sun Yat-Sen University, Guangzhou, 510006, China; 2 Pre-Incubator for Innovative Drugs & Medicine, School of Bioscience and Bioengineering, South China University of Technology, Guangzhou 510006, China; 3 Department of Immunobiology, Jinan University, Guangzhou, 510632, China; 4 Department of Cell Biology, Jinan University, Guangzhou, 510632, China

## Abstract

Mammalian target of rapamycin (mTOR) is an attractive target for new anticancer drug development. We recently developed *in silico* models to distinguish mTOR inhibitors and non-inhibitors. In this study, we developed an integrated strategy for identifying new mTOR inhibitors using cascaded *in silico* screening models. With this strategy, fifteen new mTOR kinase inhibitors including four compounds with IC_50_ values below 10 μM were discovered. In particular, compound **17** exhibited potent anticancer activities against four tumor cell lines, including MCF-7, HeLa, MGC-803, and C6, with IC_50_ values of 1.90, 2.74, 3.50 and 11.05 μM. Furthermore, cellular studies and western blot analyses revealed that **17** induces cell death via apoptosis by targeting both mTORC1 and mTORC2 within cells and arrests the cell cycle of HeLa at the G_1_/G_0_-phase. Finally, multi-nanosecond explicit solvent simulations and MM/GBSA analyses were carried out to study the inhibitory mechanisms of **13**, **17**, and **40** for mTOR. The potent compounds presented here are worthy of further investigation.

The mammalian target of rapamycin (mTOR) plays a critical role in several signaling pathways, controlling cell growth, proliferation, angiogenesis, protein translation, energy homeostasis, and lipid metabolism^1^,^2^. mTOR exists in two complexes: mTOR complex 1 (mTORC1) and complex 2 (mTORC2). The mTORC1 consists of Raptor, LST8, PRAS40 and Deptor and, regulates protein synthesis through the phosphorylation of p70S6K1 and 4E-BP1^3^. The mTORC2 consists of Rictor, LST8, SIN1, Deptor and Protor and, regulates cell proliferation and survival through the phosphorylation of Akt/PKB^4^. Aberrant activation of the mTOR signaling pathway has been commonly observed in many cancers and therefore has attracted considerable attention as an oncology drug discovery target^2^.

Rapamycin and its analogs (rapalogs) have been successfully applied to treat specific cancers in the clinic, suggesting that mTOR is a promising anticancer drug target^5^. However, recent studies have shown that existing rapalogs do not completely inhibit mTORC1 activity and have no inhibitory effect against mTORC2^6^,^7^. In addition, treatment with rapamycin and rapalogs usually results in the hyper-activation of Akt, thus reducing its benefits as an anticancer agent^8^. There is great interest in clinically testing the hypothesis that ATP-competitive mTOR inhibitors will show broad and profound anticancer activity, which may offer therapeutic advantages over rapalogs.

In recent years, ATP-competitive mTOR inhibitors, such as mTOR selective inhibitors (e.g., OSI-027^9^, INK-128^10^, and CC-223^11^) and dual mTOR/PI3K inhibitors (e.g., PF-04691502^12^, BEZ235^13^, and GSK2126458^14^) are discovered and being tested in clinical trials. These inhibitors are applied for elucidating the biochemistry of the mTOR signaling pathway, but ATP-competitive mTOR inhibitors for clinical use are not commercial available. Moreover, these inhibitors have side-effects, including skin rash, weight loss, mucositis, depression, thrombocytopaenia, and hyperlipaemia^15^,^16^. Hence, there is a continually growing need to discover novel mTOR inhibitors for further development into therapeutic candidates for cancer treatment^11^,^17^.

In the previous work, we developed an *in silico* method to predict mTOR inhibitors with multiple classification approaches including recursive partitioning (RP), naïve Bayesian (NB) learning^18^ using Atom Center Fragments (ACFs) as the features. The method has been validated for being capable of hopping new mTOR inhibitor scaffolds^18^. In this study, we continued our earlier efforts aimed at identifying and characterizing novel mTOR inhibitors. An integrated virtual screening strategy using combining multiple classification models with molecular docking approach was employed to discover new ATP-competitive mTOR inhibitors (Fig. 1). The hits selected via virtual screening were then validated using an *in vitro* mTOR kinase assay. In particular, *in vitro* anti-proliferative assay demonstrated that compound **17** exhibited potent anticancer activities against four tumor cell lines, including MCF-7, HeLa, MGC-803, and C6. The mechanisms of cell death induced by compound **17** were also probed by a series of chemical biology studies, including cell cycle analyses, quantification of apoptosis, and western blot analyses.

## Results and Discussion

### Virtual screening for mTOR inhibitors

**Figure 1:**
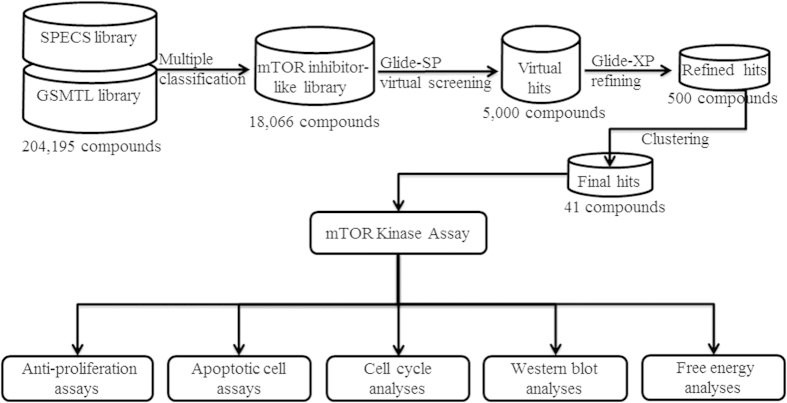
Flowchart of mTOR inhibitor discovery.

The flowchart of the virtual screening for the present study is shown in Fig. 1. In our previous study, a series of *in silico* classification models were developed for the prediction of mTOR inhibitors. In the present study, the previous multiple classification approach was employed to filter compounds in SPECS and GSMTL libraries in order to construct the mTOR inhibitor-like library. The RP model (MP+FPFP_4) was first applied for a total of 204,195 molecules and 26,596 compounds were retained. Then, the NB model (MP+LCFP_6) was employed to further filter these 26,596 compounds, resulting in 23,561 compounds. Finally, the ACFs model (ACFs layer = 3) was used to further refine these 23,561 compounds and 18,066 compounds were retained. mTOR inhibitor-like library with enhanced mTOR inhibition (18,066 compounds) was subsequently used for the virtual screening with molecular docking approach.

Prior to the virtual screening, the performance of the Glide docking was evaluated by re-docking the native ligand (PP242, PDB entry 4JT5) into mTOR kinase domain (Figure S1). As shown in Figure S1, the root mean square of distance (RMSD) between the experimental conformation of PP242 and the best conformation generated by Glide docking is 0.61 Å, suggesting that the Glide docking algorithm is qualified for docking small molecules to the mTOR active pocket. All structures from the mTOR inhibitor-like library were first docked and scored by the Glide SP score. The top 5,000 saved structures from the previous step were re-docked and scored by the Glide XP score. After this docking procedure, the 500 compounds with top Glide XP scores were stored separately for clustering and visual analyses. These compounds was inspected to check whether they had interactions with the ATP binding pocket of mTOR kinase, including hydrogen bond interactions with Val2240 and π-π stacking interaction with Trp2239. This step makes sure selected candidates not only have a higher docking score but also a rational binding mode. An *In house* S-cluster algorithm^19^ was applied for structure diversity analysis to assure the hits selected from the virtual screening were unique and unrepeated. Finally, 41 compounds were chosen for bioassay.

### *In vitro* mTOR kinase assay

The mTOR kinase inhibitory activities of the 41 final virtual hits were determined using an ELISA-based activity assays that utilize a p70S6K-GST fusion protein as a specific mTOR substrate (see the Experimental Section for details). For the initial screening, all the activities were measured at a concentration of 10 μM. The compounds were retested to exclude false positives when they showed greater than 20% inhibition of mTOR kinase activity. Detailed results of the bioassays are shown in Table 1. Among the 41 tested compounds, 15 compounds exhibited more than 20% inhibition of mTOR kinase activity and 7 compounds (**13**, **17**, **20**, **21**, **27**, **36**, **and 40**) were studied further to determine their IC_50_ values (Table 1). Dose-response curves for mTOR inhibition by active compounds are shown in Figure S2. In addition, original docking ranks and Glide docking scores are listed in Table S2. In the present study, wortmanin suggested and supplied by CBA104-1KIT from Calbiochem, which has been reported as mTOR kinase inhibitor with an IC_50_ value of 0.2 μM^20^, and under Phase IV clinical trials (also targeting PI3K kinase), was used as a positive control. Wortmanin was determined with an IC_50_ value of 0.14 μM in this study, indicating that our *in vitro* mTOR kinase assay method is both accurate and reliable. As shown in Table 1, four hits (**13**, **17**, **20**, and **40**) exhibited IC_50_ values less than 10 μM. Compound **40** showed the most potent inhibitory activity against mTOR, with an IC_50_ value of 1.47 μM. Other promising compounds were **21**, **27**, and **36**, which exhibited IC_50_ values of 14.38, 28.65 and 14.88 μM, respectively.

### Structural novelty and drug-likeness analysis of the confirmed mTOR inhibitors

**Figure 2:**
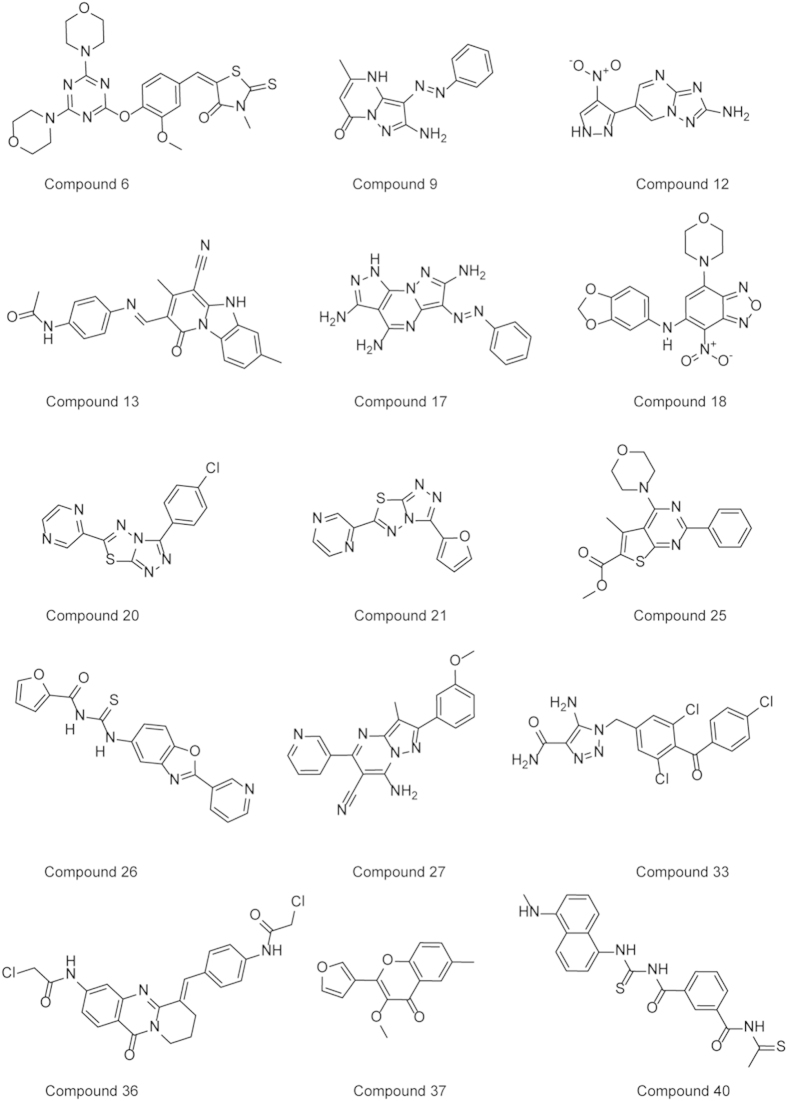
Chemical structures of the 15 confirmed active compounds.

The chemical structures of 15 active molecules are shown in Fig. 2. To evaluate the novelty of these hits with respect to known mTOR kinase inhibitors, pairwise Tanimoto similarity indices between these hits and mTOR inhibitors obtained from ChEMBL (IC_50_ < 10 μM, Figure S3)^18^ were calculated based on the FCFP_4 fingerprint via the “Find Similar Molecules by Fingerprints protocol” in Discovery Studio 3.5 (Accelrys Inc., San Diego, USA). As shown in Figure S3, these hits have low Tanimoto similarities (0.13 ~ 0.38, except 25 of 0.421) with the known mTOR inhibitors. The three most active compounds (**13**, **17**, and **40**) exhibited Tanimoto similarity values of 0.193, 0.138 and 0.346, respectively. All these results suggested that these mTOR inhibitors discovered in this study are structurally novel. In other words, the simple method (e.g., 2D similarity method) cannot discover the novel active compounds resulting from the virtual screening strategy presented in this study.

The drug-likeness properties of 15 hits were assessed using Qikprop (Table 2)^21^. As shown in Table 2, all of these inhibitors satisfied most of the drug-likeness rules defined in Qikprop. The molecular weights of the hits are less than 500 (except **6**), the number of hydrogen bond donors is fewer than 5 (except **17**) and the number of hydrogen bond acceptors is fewer than 10 (except **6**). The predicted octanol/water partition coefficient (QPlogPo/w) is in the acceptable range i.e., −2.0 to 6.5 and −6.5 to 0.5, respectively. Oral absorption index (PHOA) values of 15 hits are in the acceptable range (Table 2). Moreover, active compounds presented in this study (except compound 6) don’t contain problematic substructures suggested from Pan Assay Interference Compounds (PAINS)^22^ based on substructure search results. Although compound 6 contains rhodanine substructure from PAINS, it shows low activity against mTOR (Table 1). All of these results suggested that the novel mTOR inhibitors identified in the present study provided valuable alternatives for further lead optimization.

### Characteristic binding patterns of the confirmed hits

**Figure 3:**
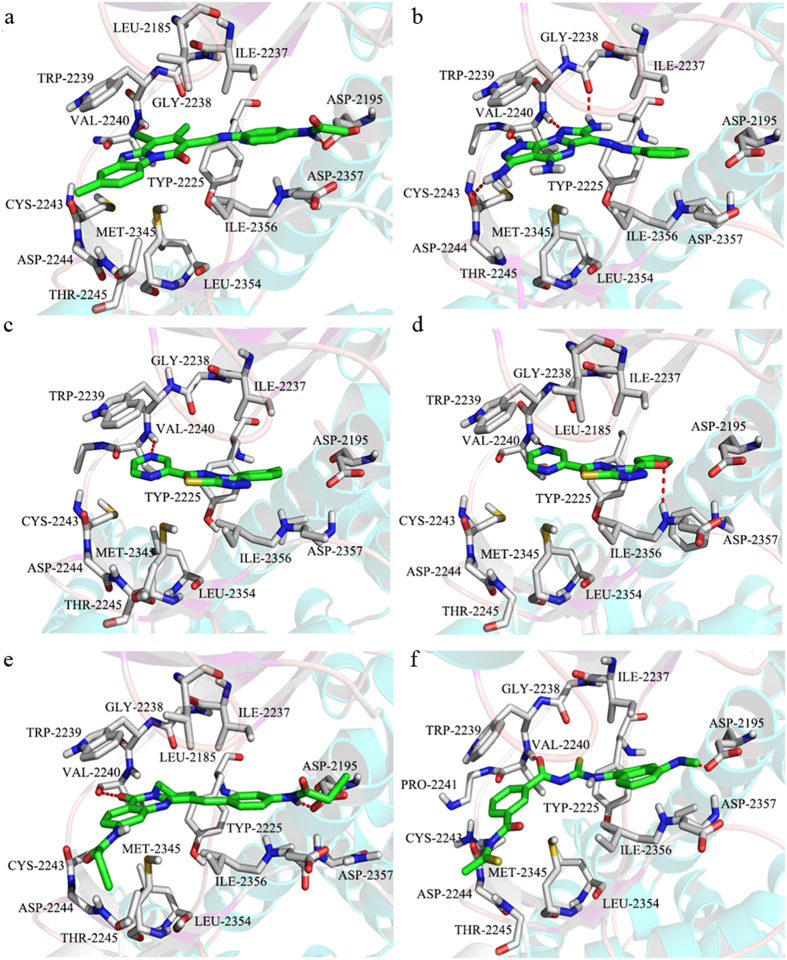
Predicting binding modes for compounds 13 (**a**), 17 (**b**), 20 (**c**), 21 (**d**), 36 (**e**), and 40 (**f**). Hydrogen bonds are depicted by red dotted lines. These figures were prepared using PyMol software (http://www.pymol.org/).

**Figure 4:**
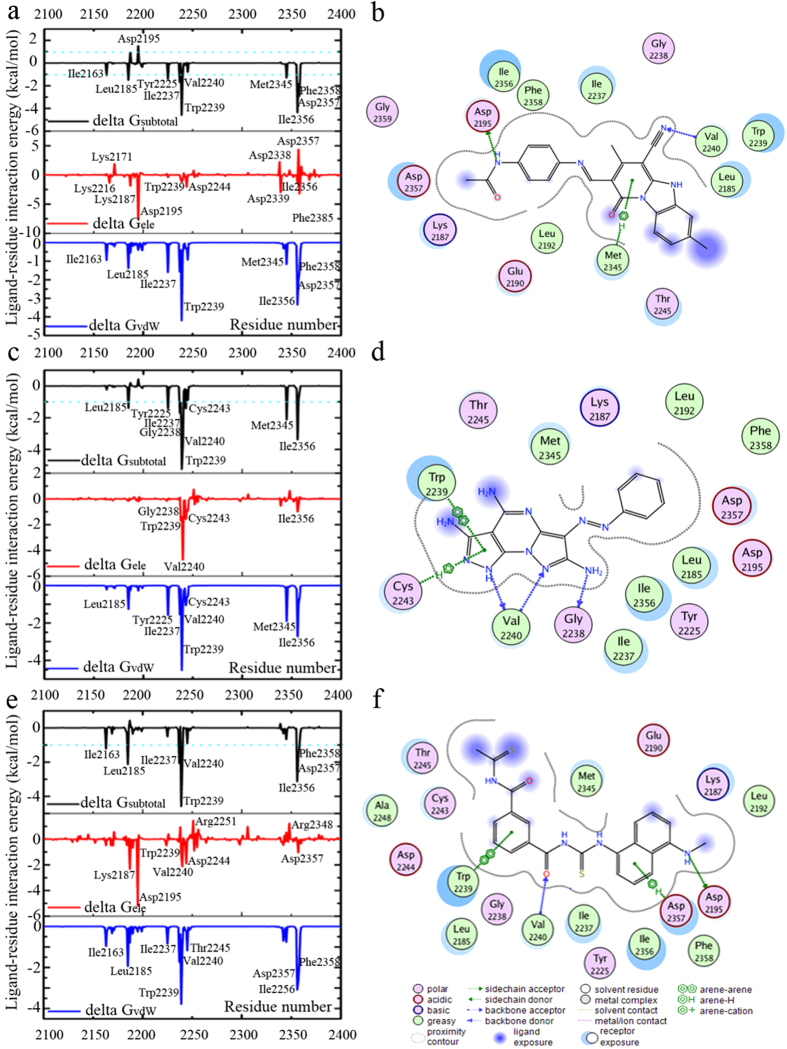
Ligand−residue interaction energies from MM/GBSA energy decomposition for 13 (**a**), 17 (**c**) and 40 (**e**), and two-dimensional ligand-residue interaction maps of 13 (**b**), 17 (**d**) and 40 (**f**). Delta G_subtotal_ represents total estimated binding free energy for each residue. Delta G_ele_ represents non-bonded electrostatics interactions. Delta G_vdw_ represents non-bonded van der Waals interactions.

The binding modes of six inhibitors (**13**, **17**, **20**, **21**, **36**, and **40**) with mTOR were predicted by molecular docking. Subsequently, the energy profile and stability of the predicted binding poses of three most potent inhibitors (**13**, **17**, and **40**) were investigated through MD simulations and MM-GBSA calculations. **13** (IC_50_ = 5.83 μM) represents a unique chemotype that consists of ternary rings, linker, and benzene acetamide. The ternary rings are surrounded by Val2240, Trp2239, Met2345, Leu2185, Leu2354, and Thr2245 (Figs 3a and 4a), while benzene acetamide is inserted in a deep pocket containing Asp2195, Asp2357, Phe2358, and Lys2187. **13** can form hydrogen bonds with the side chains of Val2240 and Asp2195 (Figs 3a and 4b). Distance analyses suggest that the hydrogen bond between **13** and the side chain of Val2240 is conserved during 10 ns MD simulations (Figure S4a). Previous studies demonstrated the hydrogen bond between inhibitor and Val2240 was necessary for mTOR inhibitory activity^23^,^24^,^25^, which was consistent with our analysis. The hydrogen bond between **13** and the carboxyl oxygen atom of Asp2195 is maintained for the first 5 ns in our simulations and occasionally disappears after 5 ns (Figure S4b), suggesting this hydrogen bond is not stable in water. Moreover, **13** can form extensive stacking and arene-H interactions with the indole group of Trp2239 (Fig. 3a) and the side chain of Met2345 (Fig. 4b). The binding free energy was estimated using the MM/GBSA method to gain information on the different components of interaction energy that contributes to **13** binding, and detailed results are listed in Table S3. Both van der Waals and electrostatic components play key roles in **13** binding, and the van der Waals contribution (−53.44 kcal/mol) is approximately 2-fold greater than the electrostatic component (−24.14 kcal/mol). Electrostatic solvation (ΔG_ele,solv_) disfavors binding because of the de-solvation penalty for **13** and mTOR. The non-polar component of solvation (ΔG_nonpol,solv_), which corresponds to the burial of solvent-accessible surface area (SASA) upon binding, provides a slightly favorable contribution (Table S3). Energy decomposition analysis led to the identification of key residues that contribute to binding affinity at the active site. Generally, if the interaction energy between the residue and inhibitor is lower than –1 kcal/mol, that residue is considered to be important in inhibitor binding (i.e., it is hot residue)^26^,^27^. As shown in Fig. 4a, hot residues can be divided into three clusters for **13** binding, i.e., (i) “hydrophobic chamber” (residues Ile 2163, Trp2239, Val2240, and Met2345), (ii) “inner hydrophobic pocket” (residues Ile2237, Leu2185, Tyr2225, and Ile2356), and (iii) “deeper pocket” (residues Asp 2357 and Phe2358). The major contribution for each hot residue is from van der Waals interactions (Fig. 4a). Our energy decomposition analyses results are consistent with the binding mode results. For Asp2195, the electrostatic contribution (−7.72 kcal/mol) is in favor for **13** binding, but electrostatic solvation (ΔG_ele,solv_, 9.82 kcal/mol) is unfavorable for **13** binding, resulting in an overall unfavorable contribution from Asp2195 (ΔG_subtotal_ = 1.51 kcal/mol, Fig. 4a). This result also agrees with the unstable hydrogen bond between phenyl acetamide group of **13** and the carboxyl oxygen atom of Asp2195 (Figure S4a). It is quite possible that **13** possesses a relatively polar of acetamide group that cannot form sufficient interactions with Asp2195.

The predicted binding mode of **17** and mTOR is shown in Figs 3b and 4d. **17** can form three hydrogen bonds with the side chains of Val2240 and Gly2238, and these hydrogen bonds are maintained over the course of 10 ns MD simulations (Figure S4b). Similar to **13**, the pyrimidine group of **17** also forms an arene-arene stacking interaction with the indole group of Trp2239 (Figs 3b and 4d). Previous studies^23^,^28^ suggested that Trp2239 is not present in canonical protein kinases (e.g., PI3Ks); it would contribute to the inhibitory specificity for mTOR over PI3Ks. Our western blot analyses demonstrate that **17** cannot inhibit the PI3Ks pathway (See cellular assays section). All of these results suggest that if an inhibitor can form stronger interaction with Trp2239 (especially arene-arene stacking interactions, e.g., ΔG_subtotal_ = −4.61 kcal/mol for **17**, Fig. 4b), it may be a selective mTOR inhibitor. Additionally, an arene-H stacking interaction is formed between the pyrimidine group of **17** and the side chain of Cys2243 (Fig. 4b). Residue-based free energy analyses suggest that the hot residues for **17** binding are similar to the hot residues in **13** (Fig. 4c), except for Asp2357 and Phe2358. It is possible that there are no any substituents in the Benzene group of **17** which cannot reach the “deeper pocket” to interact with Asp2357 and Phe2358 compared with **13**. This may explain why the binding free energy of **17** is relatively higher than **13** (Table S3). All of these results are consistent with the bioassay results (Table 1).

**40** exhibited the most potent inhibitory activity against mTOR, with an IC_50_ value of 1.47 μM. As shown in Fig. 3f and 4f, **40** forms all of the favorable interactions exhibited in **13** and **17**, i.e., (i) hydrogen bonds with the side chain of Val2240 (conserved during MD simulations, Figure S4c), (ii) arene-arene stacking interactions with the indole group of Trp2239, and (iii) similar hot residues with **13** for **40** binding (Fig. 4e). Similar to **13**, the hydrogen bond between **40** and the carboxyl oxygen atom of Asp2195 is not stable (Figure S4c). Moreover, additional arene-H stacking interaction is observed between the benzene group of **40** and the side chain of Asp2357. All of these favorable interactions suggest the binding free energy of **40** is lower than that of **13** and **17 (**Table S3), which also agree with the trend of our experimental observation. **20** and **21** show similar binding modes, while **36** exhibits a similar binding mode with **13**. Detailed binding modes of **20**, **21**, and **36** are depicted in Fig. 3c–e.

### Cellular assays

**Figure 5:**
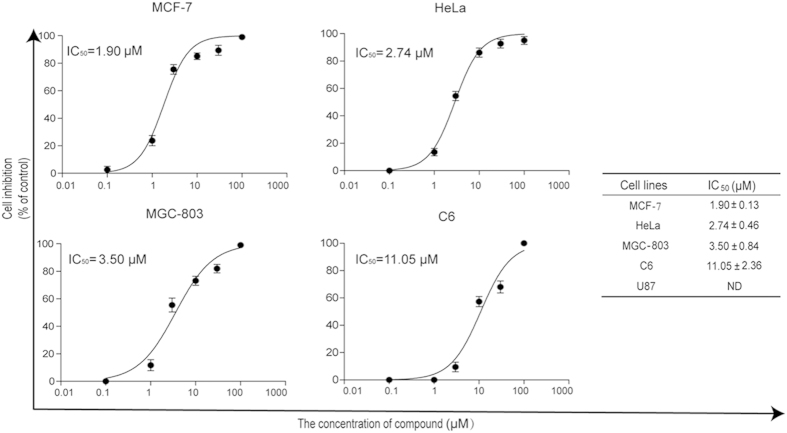
*In vitro* anti-proliferative activity of **17**. The relative inhibition rates of anti-proliferative activity were determined by MTT assays. The highest concentration of **17** is 100 μM. IC_50_ values were determined from the results of at least three independent tests of four cell lines.

The three most potent compounds (**13**, **17**, and **40**) were selected for *in vitro* anti-proliferative assays using five cancer cell lines from different tissues (Fig. 5), including MCF-7 (human breast cancer cell), HeLa (human cervical cancer cell), MGC-803 (human gastric cancer), C6 and U87 cells (Glioma cell). **13** and **40** do not show anti-proliferative activity against these cancer cell lines (data not shown), which may be due to the effect of a drug efflux pump^21^,^28^. **17** exhibits inhibitory effects on the proliferation of MCF-7, HeLa, MGC-803, and C6 in a dose-dependent manner, with IC_50_ values of 1.87, 2.74, 3.51 and 10.19 μM, respectively (Fig. 5). Most human breast cancer cells and cervical cancer cells become resistant to current chemotherapeutic drugs due to mutation of apoptotic mechanisms^29^,^30^. It has been reported that MCF-7 cells are resistant to most of the drugs approved by the US Food and Drug Administration (FDA), including paclitaxel, doxorubicin, 5-fluorouracil, etoposide, and camptothecin^31^,^32^. Kinase profile assay results suggest that compound 17 is multiple kinase inhibitors (mTOR, DNA-PK, and p110 alpha, Table S4), suggesting it significantly exhibits anticancer activities in cell lines in the present study. Anti-proliferative assay results demonstrate that **17** can be a promising lead for further development into therapeutic candidates for treatment of these cancers.

**Figure 6:**
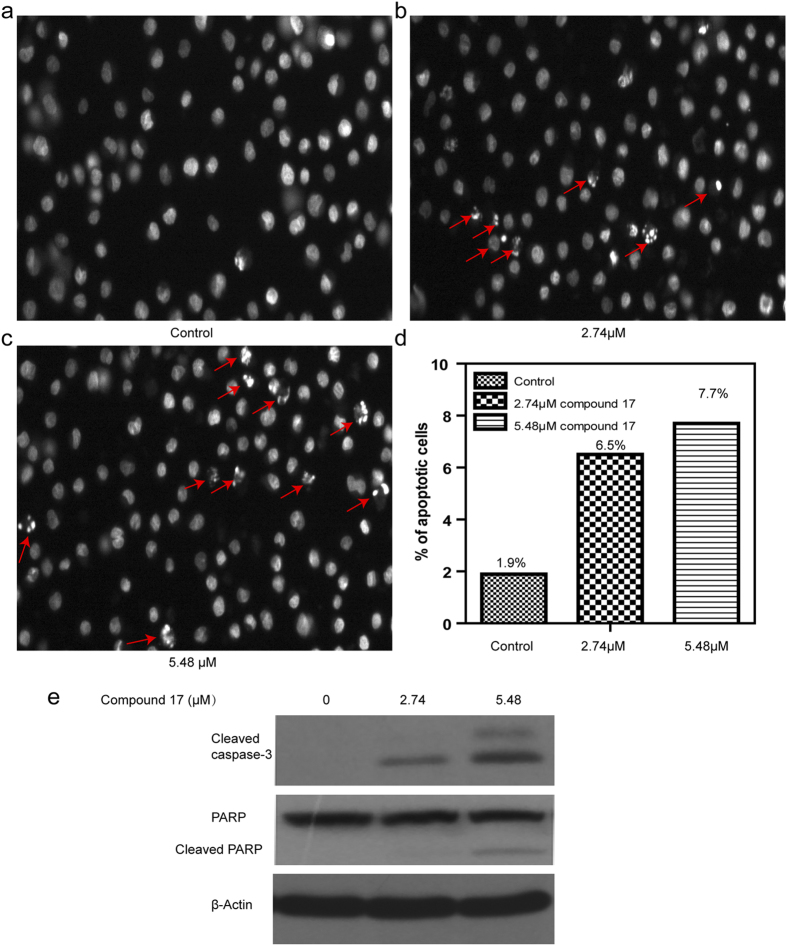
17 induced apoptotic cell death in HeLa cells. Representative nuclear staining of HeLa cells with Hoechst 33342. Changes in cellular nuclear morphology examined using fluorescence microscopy. (**a**) Control cells uniformly strained blue without condensed chromatin, with normal, round, and unpunctuated nucleus, recorded as non-apoptotic. Cells incubated with **17** at concentrations of (**b**) 2.74 μM, (**c**) 5.48 μM for 24 hours. **17**-treated cells with condensed and fragmented nuclei (red arrow) were scored as apoptotic cells compared with the control. (**d**) Quantitative of apoptosis in HeLa cells. (**e**) Western blotting showing the activation of capase-3 cleavage of PARP in cell treated of compound 17 for 24 h. Representative plots of three independent experiments was performed. ***P < 0.001 versus control.

To elucidate the mechanisms of cell death induced by **17**, we analyzed the effect of **17** on nuclear morphology using Hoechst 33342 staining. Detailed results are shown in Fig. 6. We observed that the nuclei of compound **17**-treated HeLa cells exhibited hyper-condensed chromatin or shrunken and fragmented nuclei. This observation allowed for clear discrimination of treated cells from untreated non-apoptotic HeLa cells, which have a normal, round, and unpunctuated nucleus. These results demonstrated that the mechanism of cell death induced by compound **17** was via apoptosis. The percentage of apoptotic HeLa cells were 6.5% and 7.7% at the concentrations of 2.74 and 5.48 μM, respectively (Fig. 6d). To further confirm the nuclear morphology observation, Western blotting was used to detect caspase-3 activation and PARP cleavage, two biochemical markers of apoptosis. Compound 17 markedly increased the levels of cleaved (activated) caspase-3 and PARP compared with control (Fig. 6e). The induction of apoptosis is positively correlated with the concentration of compound **17**.

**Figure 7:**
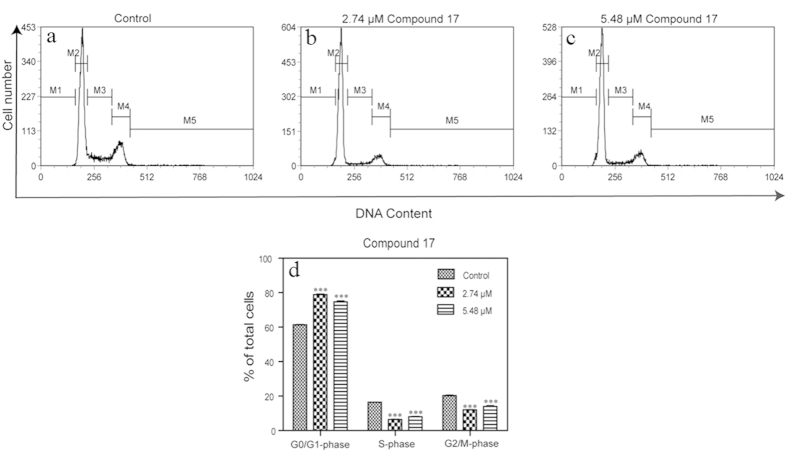
Effects of compound **17** on the distribution of cell cycle of HeLa cells. (**a**) Control cells without the treatment of compound **17**. Cells incubated with compound **17** at concentration of (**b**) 2.74 μM, (**c**) 5.48 μM for 24 hours. (**d**) Quantitative measurement of the distribution of cell cycle in HeLa cells. M1: sub-G_1_/G_0_; M2: G_1_/G_0_; M3: S; M4: G_2_/M. Representative plots of three independent experiments were performed. ***P < 0.001 versus control.

To further explore the mechanism of cell growth inhibition by compound **17**, we examined the effects of compound **17** on cell cycle distribution in HeLa cells using flow cytometry. As shown in Fig. 6, the G2/M-phase and S-phase were reduced compared with controls, while the G_1_/G_0_-phase was increased (Fig. 7). These results demonstrated that **17** induced an arrest of cell cycle at the G_1_/G_0_-phase, contributing to its anti-proliferative effect on cancer cells.

**Figure 8:**
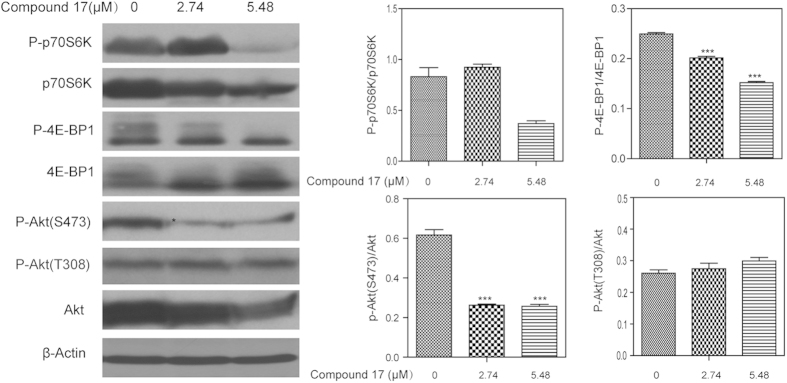
Effects of compound **17** on the mTORC1, mTORC2, and AKT activity in HeLa cells by Western blotting. Inmunoblot analysis of phosphorylation blocking effects of **17** for p70SK6, 4E-BP1, Akt (S473), and Akt (T308) (Left). Quantitative measurement of the phosphorylation levels of these proteins (Right). *β*-actin was used as a loading control. *P <0.05, and ***P <0.001 versus control.

*In vitro* mTOR kinase assay confirmed that **17** did inhibit mTOR kinase activity (Table 1). The mTOR pathway is frequently activated in human cancers (e.g., HeLa and MCF-7). To assure compound **17** inhibits the mTOR pathway inside the cell, western blotting was employed to check the activity of mTORC1 (Complex 1) and mTORC2 (Complex 2). HeLa cell line was selected for western blot analysis after treatment with compound **17** at 2.47 and 5.48 μM; detailed results are shown in Fig. 8.

mTORC1 activity was evaluated by determining the phosphorylation level of its targets, p70S6K and 4E-BP1, in the HeLa cell line. As shown in Fig. 8, the phosphorylation levels of p70S6K and 4E-BP1 were suppressed by the treatment of compound **17** at 2.47 and 5.48 μM, respectively. Quantitative of the phosphorylation levels of p70SK6 and 4E-BP1 are positively correlated with the concentration of **17** (Fig. 8). These results demonstrated that mTORC1 was well inhibited inside HeLa cells by **17**.

Previous studies demonstrated that the kinase domains of mTORC1 and mTORC2 exhibited similar 3D structures, especially in the ATP binding pocket^17^,^23^,^25^,^33^. Accordingly, we checked mTORC2 activity by measuring Akt phosphorylation levels on Ser473 in HeLa cells. As shown in Fig. 8, compound **17** can reduce P-Akt Ser473, suggesting that mTORC2 is well inhibited inside the cell of HeLa. In other words, the mechanism of HeLa cell death induced by **17** was apoptosis, and induction was achieved by targeting both mTORC1 and mTORC2 simultaneously.

To evaluate whether compound **17** inhibits the PI3K pathway, the Akt phosphorylation level on Thr308 was determined. Figure 8 suggested that P-Akt Thr308 (the PDK1 target, a downstream kinase, in the PI3K pathway) was not affected by treatment with **17** compared with the controls. All of these results demonstrated that compound **17** is a selective mTOR inhibitor.

Accordingly, the novel mTOR inhibitors identified in the present study provided valuable alternatives for further development into therapeutic candidates for cancer treatment. The development of derivatives from several of these potent compounds (especially compound **17**) is currently under investigation.

The study aims to discover hits with new scaffold against mTOR using virtual screening method. This goal had been achieved. The parallel signaling (e.g., MEK/ERK and STAT3/5) of TOR KD or KO cells inhibited by compound **17** will be examined in future study.

## Methods

### mTOR inhibitor like library and virtual screening

Two virtual libraries, SPECS (197,116 compounds)^34^ and GSMTL (Guangdong Small Molecule Tangible Library, 7,079 compounds)^35^, were selected to construct a mTOR inhibitor like library. In the libraries, all compounds containing inorganic atoms were removed prior to any processing. Then, all the structures of the compounds were chemically standardized (including adding hydrogen atoms, ionizing at the pH range from 5.1 to 9.1, and generating stereoisomers and valid single 3D conformers) by means of the LigPrep module in Maestro (version 9.4, Schrödinger).

Three classifiers (RP model: MP+FPFP_4, NB model: MP+LCFP_6, and ACFs model: mTOR Predictor) were employed to derived the mTOR inhibitor-like library from SPECS and GSMTL.

The co-crystal structure of mTOR with PP242 (PDB entry 4JT5)^23^ was used as the structural template for virtual screening with molecular docking approaches. The template was manipulated with the “Protein Preparation Wizard” workflow in Maestro. The main manipulations are removing all water molecules, protonation, and optimization based on OPLS_2005 force field. Then, a docking grid was generated using the “Receptor Grid Generation” module of Maestro. The grid encloses a box centered on the native ligand with a dimension of 10 × 10 × 10 (*x* × *y* × *z*, Å). The scaling factor of 0.8 was set for van der Waals radii of receptor atoms with a partial atomic charge less than 0.15.

Standard precision of Glide docking procedure (Glide-SP) was employed to screen the mTOR inhibitor-like library. For each compound, up to 50,000 conformations were generated for docking. The best docking pose of the compound was kept based upon Glide scoring function (G-score). Thus, the compounds in the mTOR inhibitor-like library were sorted with G-score. Then, the top 5,000 ranked compounds were re-docked and scored with Glide extra precision (Glide-XP), which performs the conformational sampling more rigorously and uses a refined scoring function. Thus, the top 5,000 compounds were re-sorted by Glide-XP. Again, the top 500 compounds were selected from the 5,000 re-ranked compounds for clustering. Clustering and visual analyses were carried out to remove redundancies resulting from similar structures and, check the docking poses and interactions between the ligands and receptor. Finally, 41 compounds were selected and purchased for bioassays.

### Chemistry

All compounds tested for mTOR inhibitions were acquired from the SPECS and GSMTL. Compound purity was assessed by HPLC equipped with a XDB-C18 column (250 mm × 4.6 mm, 5 μm particle size) and a UV/VIS detector setting of λ = 254 nm. Compounds were eluted with the two solvent systems (CH_3_OH as the organic phase in method I and CH_3_CN as the organic phase in method II). HPLC analysis of the compounds assayed confirmed the purity to be ≥95% (Table S1). ^1^H-NMR and MS spectra data were recorded on a Bruker AvanceIII spectrometer at 400 MHz using TMS as reference (Bruker Company, USA) and Agilent 6120 using methanol as solvent(Agilent, German), respectively. Detailed results can be found in Figure S7.

### *In vitro* mTOR kinase inhibition assay

mTOR activity was evaluated using a purified recombinant mTOR fragment (amino acids 1360-2549, CBA104-1KIT from Calbiochem). The K-LISA^TM^ mTOR activity kit is an ELISA-based activity assay for detection of phosphorylation of a p70S6K-GST fusion protein (a specific mTOR substrate) in the presence of ATP. The assay was optimized for use with mTOR as described in the Calbiochem protocol. The assay takes place in 96-well plates and can be divided into two phases: an mTOR kinase reaction phase and phosphorylated substrate detection phase.

In the kinase reaction phase, the following components were mixed in the same well: 5 μL of a solution containing 5 μg/mL mTOR substrate (recombinant p70S6K-GST fusion protein) in Tris-buffered saline (1 L TBS: sodium chloride 8.8 g, Tris Base 2.4 g, HCl 13 mL, Tween-20 10 ml, ddH_2_O), 5 μL ATP solution containing 20 mM ATP diluted in kinase assay buffer (pH 7.4, 0.01% Tween 20, 1 mM EDTA, 10 mM MgCl2, 1 mM DTT), 65 μL of human recombinant mTOR kinase at the optimal concentration (80 ng/well) diluted in kinase assay buffer, and 5 μL of serial dilutions of inhibitors diluted in DMSO and kinase assay buffer. For each inhibitor concentration, the assay was run in triplicate. The final reaction volume was 100 μL per well, and samples incubated for 30 min at room temperature.

The kinase reaction was stopped by adding 10 μL kinase stop solution to each well. The phosphorylated substrate was detected using Anti-p70S6K-pT389 antibody, followed by detection with HRP-Antibody Conjugate and TMB Substrate. Then, ELISA stop solution was added to each well to stop the reaction, and relative mTOR inhibition activity was determined by reading the absorbance at dual wavelengths of 450/540 nm. To exclude any possible nonspecific/promiscuous or artificial inhibition of mTOR, all mTOR kinase assays are repeated in the presence of 0.01% Triton X-100, as suggested by Shoichet and coworkers^36^,^37^. None of the observed inhibitory activities was affected by the addition of the nonionic detergent, confirming the active compounds are reliable.

### *In vitro* cell proliferation assay

Cell proliferation was measured with an MTS assay (CellTiter 96 Aqueous ONE Solution kit; Promega, Madison, USA). MCF-7, HeLa, MGC-803, C6 and U87 cells in log-phase were seeded (5 × 10^3^ cells/well) in 96-well plates for 24 h, and then the medium were replaced with fresh medium containing different concentrations of compounds. The working concentration of DMSO did not exceed 0.2%. After incubation for 48 h, the cells were further incubated with 20 μL of MTS for 1–4 h at 37 °C in a humidified incubator with 5% CO_2_. The absorbance at 490 nm was measured using a mircoplate reader (Model680, Bio-Rad; Hercules, CA, USA). The inhibition rate (%) was calculated as equation (1):


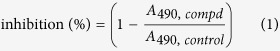


IC_50_ values were determined from the results of at least three independent tests and calculated from the inhibition curves.

### Cell cycle analysis

Cells cycle distribution was analyzed as previously described^38^. In brief, HeLa cells (1.5 × 10^4^/well) seeded in six-well plates were incubated with compound **17** for 24 h. Then, the cells were collected, washed twice with phosphate-buffered saline (PBS), and fixed in 70% cold ethanol at −20 °C overnight. Finally, the samples were stained with 50 μg/mL propidium iodide and 30 μg/mL of RNase A at 37 °C for 1 h. DNA content data were acquired using CELLQuest software on a flow cytometer (FACSCalibur; Becton Dickinson, Mountain View, CA, USA).

### Quantification of apoptotic cells

HeLa cells (1.5 × 10^4^/well) were grown in the presence of compound **17** for 48 h. Then, the cells were collected, washed with PBS, and fixed in 4% paraformaldehyde for 15 min at room temperature. Finally, the samples were stained with Hoechst33342 (10 μg/mL) for 5 min. Stained nuclei were photographed under a fluorescence microscope with a 20× objective (Nikon).

### Western blot analysis

HeLa cells were cultured in a six-well plate in the presence of compound **17** for 48 h. Samples were prepared by lysing PBS-washed cells with RIPA buffer (Beyotime, Haimen, China) consisting of 1% Nonidet P-40, 0.5% sodium deoxycholate, 0.1% SDS, and 50 mM Tris-HCl, pH 7.4, supplemented with NaF, NaVO_4_, EDTA and a protease inhibitor cocktail (Roche). Proteins were subjected to SDS-PAGE gel electrophoresis and transferred onto a polyvinylidene difluoride membrane (Hybond-P; GE Healthcare Life Sciences, Piscataway, NJ). Immunoblotting was performed using rabbit monoclonal antibodies (Cell Signaling Technology; Danvers, MA) against p70S6K1 phosphorylated on Thr 389 (#9234) and total p70S6K1 (#2708), 4E-BP1 phosphorylated on Thr 37 (#2855) and total 4E-BP1 (#9452), Akt phosphorylated on Thr 308 (#4056) or Ser 473 (#4060) and total Akt (#4685), cleaved caspase-3 (#9664), PARP (#9532), and *β*-actin (#3700). Bands were revealed with enhanced chemiluminescence kit (BeyoECL Plus; Beyotime) and recorded on X-ray films (Kodak; Xiamen, Fujian, China). The densitometry of each band was quantified by FluorChem 8000 (AlphaInnotech, San Leandro, CA).

### Kinase profiling assay

To characterize the kinase selectivity of compound **17**, we profiled its ability to bind to a panel of 24 structurally related kinases using the SelectScreen Kinase Profiling Service (Life Technologies Corporation, Madison, WI).

### Statistical analysis

Statistical analysis was performed using GraphPad Prism 4.0 (GraphPad Software Inc., San Diego, CA). Data are presented as the mean ± SD from at least three independent experiments, and differences are considered significant when *P* values < 0.05 as determined by the unpaired Student *t* test.

### Molecular dynamics (MD) simulations

MD simulations were carried out to investigate the binding patterns of the three inhibitors (**13**, **17** and **40**) that showed the best inhibitory potency against mTOR. The docked structures of inhibitors in complex with mTOR were used as the initial coordinates for MD simulations. The protein and inhibitors were applied with ff99SB^39^ and Generalized Amber Force Field (GAFF)^35^,^40^ force field, respectively. Each system was solvated in a truncated octahedron box of TIP3P water molecules with a margin distance of 10 Å. Neutralizing counter ions were added to the simulation system. A production simulation run for 10 ns was performed using the NPT ensemble under a target temperature of 310 K and a target pressure of 1 atm. Coordinate trajectories were saved every 1 ps for the whole MD runs. MD simulations were performed in AMBER 12^41^. A detailed preparation of inhibitors and mTOR protein as well as MD simulations can be found in the Supplementary Information.

### Binding free energy analysis

To provide insight into the interaction energies and energetic stabilities of the complexes and the contribution of each residue to inhibitor binding, the MM/GBSA method^42^,^43^,^44^,^45^,^46^ in the AMBER 12 suite was used to calculate the binding free energies for three inhibitors (**13**, **17** and **40**), and then binding free energy was decomposed into individual residue contributions. Detailed calculations and analyses can be found in the Supplementary Information.

## Additional Information

**How to cite this article**: Wang, L. *et al.* Discovering new mTOR inhibitors for cancer treatment through virtual screening methods and *in vitro* assays. *Sci. Rep.* 6****, 18987; doi: 10.1038/srep18987 (2016).

## Supplementary Material

Supplementary Information

## Figures and Tables

**Table 1:** Virtual screening hits and their *in vitro* assay results for mTOR inhibitions.

Compound	% of mTOR inhibition at 10 μM^a^	IC_50_(μM)^b^
6	22%	ND
9	23%	ND
12	30%	ND
13	76%	5.83 ± 1.85
17	61%	7.59 ± 2.44
18	38%	>50
20	55%	9.20 ± 2.15
21	50%	14.38 ± 2.02
25	36%	>50
26	33%	>50
27	38%	28.65 ± 2.65
33	21%	ND
36	31%	14.88 ± 1.01
37	22%	ND
40	91%	1.47 ± 0.33
Wortmanin^c^	–	0.14 ± 0.02

^a^% Inhibition values are the mean ± SD of triplicate measurements at 10 μM.

^b^IC_50_ values for mTOR shown are the mean ± SD of triplicate measurements.

^c^Used as a positive control compound. ND: not determined. Wortmanin is suggested and supplied by CBA104-1KIT from Calbiochem.

**Table 2:** Assessment of drug-like properties of the lead molecules and wortmanin as verified by Qikprop.

Compound	MW^a^	HD^b^	HA^c^	QPlogPo/w^d^	PHOA^e^
6	530.62	0	11.15	3.89	86.15
9	268.28	3	6.50	1.04	76.51
12	246.19	3	6.50	−0.93	42.63
13	397.44	2	8.00	2.60	78.29
17	310.32	7	7.00	−0.49	36.24
18	385.34	1	8.20	1.35	64.90
20	314.75	0	5.50	2.39	93.30
21	270.27	0	6.00	1.17	85.13
25	369.44	0	6.20	3.54	100.00
26	364.38	1	6.00	3.46	100.00
27	356.39	2	6.25	2.85	88.63
33	424.67	3	6.00	2.94	77.19
36	471.34	2	9.00	3.80	95.70
37	256.26	0	3.75	2.78	100.00
40	436.55	3	5.50	5.13	100.00
Wortmanin^f^	428.44	3	13.40	0.31	39.30

^a^Molecular weight (256.26–530.62).

^b^Hydrogen bond donors (<5).

^c^Hydrogen bond acceptors (<10).

^d^Predicted octanol/water partition co-efficient logP (recommended range: −2.0 to 6.5).

^e^Percentage of human oral absorption (<25% is poor and >80% is high).

^f^Used as a positive control compound.
