# Supplementary material for: Discovering new mTOR inhibitors for cancer treatment through virtual screening methods and in vitro assays
Source: Sci Rep. 2016 Jan 6;6:18987. doi: 10.1038/srep18987 (PMC4702177; doi:10.1038/srep18987)
Supplement: Supplementary Information [file srep18987-s1.doc]

**Supplementary Information**

**Discovering new mTOR inhibitors for cancer treatment through virtual screening methods and in vitro assays**

Ling Wang^1^,^2‡^, Lei Chen^1,‡^, Miao Yu^1^, Li-Hui Xu^4^, Bao Cheng^1^, Yong-Sheng Lin^1^, Qiong Gu^1^, Xian-Hui He^3^,* & Jun Xu^1^,*

^1^Research Center for Drug Discovery & Institute of Human Virology, School of Pharmaceutical Sciences, Sun Yat-Sen University, Guangzhou, 510006, China

^2^Pre-Incubator for Innovative Drugs & Medicine, School of Bioscience and Bioengineering, South China University of Technology, Guangzhou 510006, China

^3^Department of Immunobiology, Jinan University, Guangzhou, 510632, China

^4^Department of Cell Biology, Jinan University, Guangzhou, 510632, China

| **Contents**  **Figure S1:** Performance of the Glide docking for mTOR target.  **Figure S2:** Dose-response curves for the mTOR inhibitions of **13**, **17**, **20**, **21**, **27**, **36**, and **40**.  **Figure S3:** The closest compounds from ChEMBL to the confirmed mTOR inhibitors in the present study. All calculations were done in Discovery Studio 3.5. 2D-similarity calculations.  **Figure S4:** Time dependence of distance-based hydrogen bond analysis for **13**, **17** and **40** during 10 ns MD simulations. |  |
| --- | --- |
| **Figure S5:** Time dependence of RMSD plot for the backbone atoms of **13**, **17**, and **40**-mTOR complexes during 10 ns MD simulations. |  |
| **Figure S6:** Time dependence of RMSD plot for the backbone atoms of **13**, **17** and **40**-mTOR complexes during 10 ns MD simulations.  **Figure S7.** ^1^H NMR and MS data of selected compounds  **Figure S8.** ^1^H NMR spectra of 17 supplied by SPECS Inc. |  |
| **Table S1:** HPLC analysis data  **Table S2:** The corresponding number, docking score, source of database, ID and the ranks in Table 1 of the virtual screening hits after clustering for bioassays. |  |
| **Table S3:** Free energy analysis for the binding of **13**, **17**, **40** to mTOR. |  |
| MD simulations and Binding free energy analysis.  **Table S4:** Percentages of enzymatic inhibitions by compound **17** (10 μM) on 24 structurally related kinases of mTOR. |  |


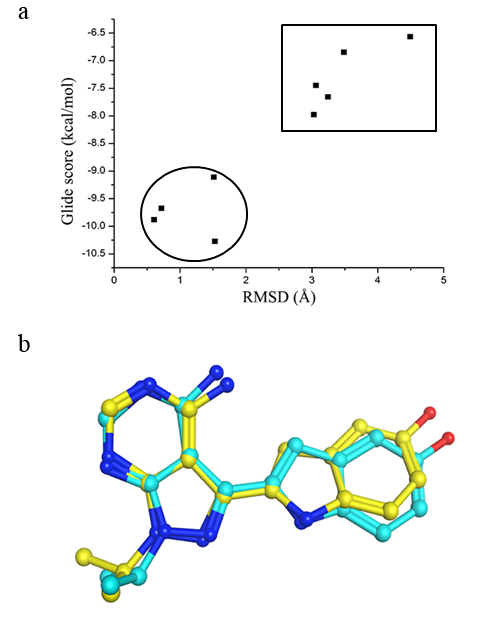


**Figure S1.** Performance of the Glide docking for mTOR target. (a) The RMSD value between experiment active conformation (PP242) and predicted conformations generated from Glide docking method are correlated with Glide score. The oval represents the good conformations obtained from Glide docking with good Glide score and lower RMSD values. The rectangle represents the bad conformations obtained from glide docking with bad glide score and larger RMSD values. (b)The best conformation generated from glide docking can map well with experimental PP242 active conformation. Yellow represents experimental PP242 active conformation and cyan represents theoretical PP242 conformation generated from Glide docking.


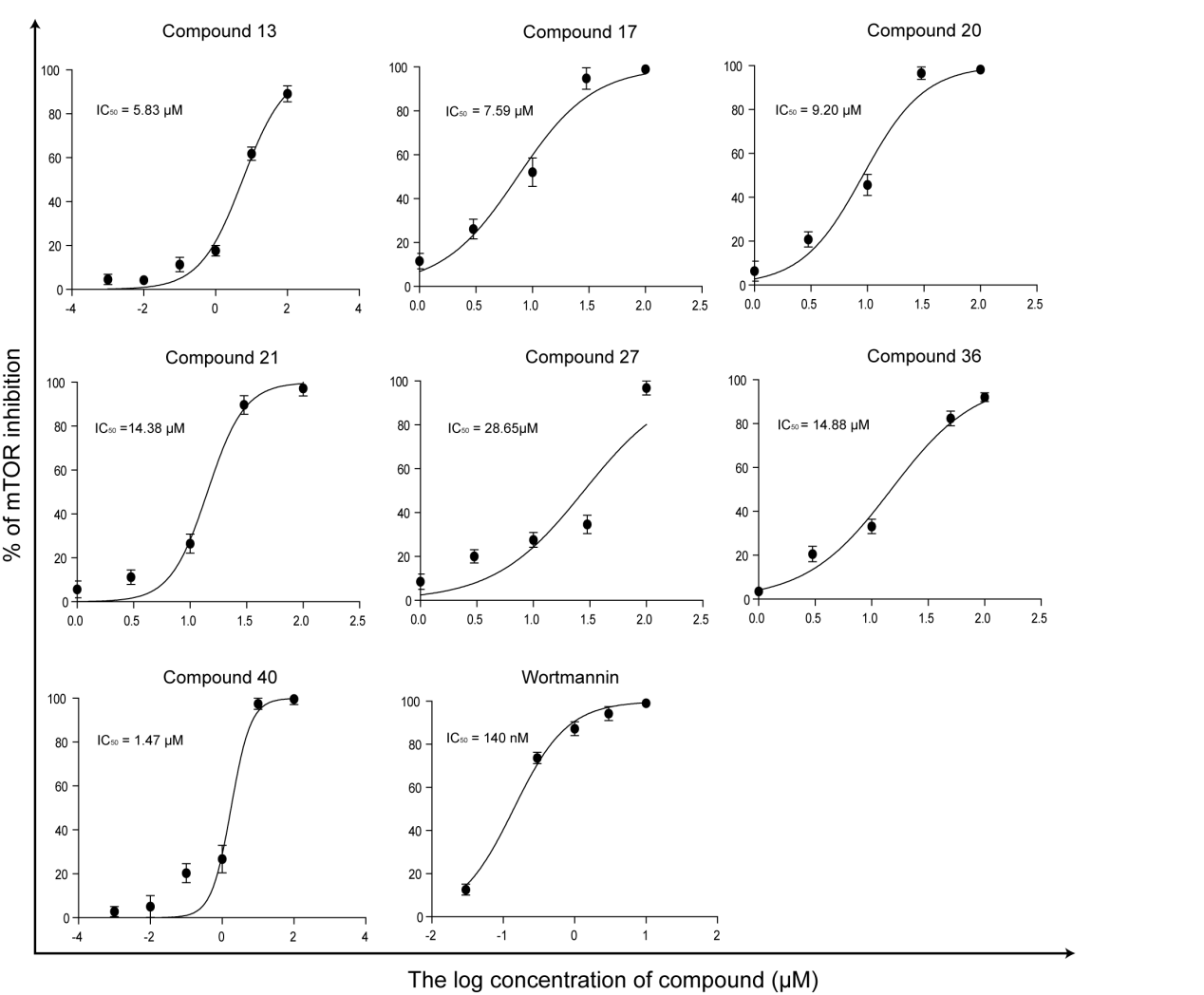


**Figure S2.** Dose-response curves for the mTOR inhibitions of **13**, **17**, **20**, **21**, **27**, **36**, and **40**.


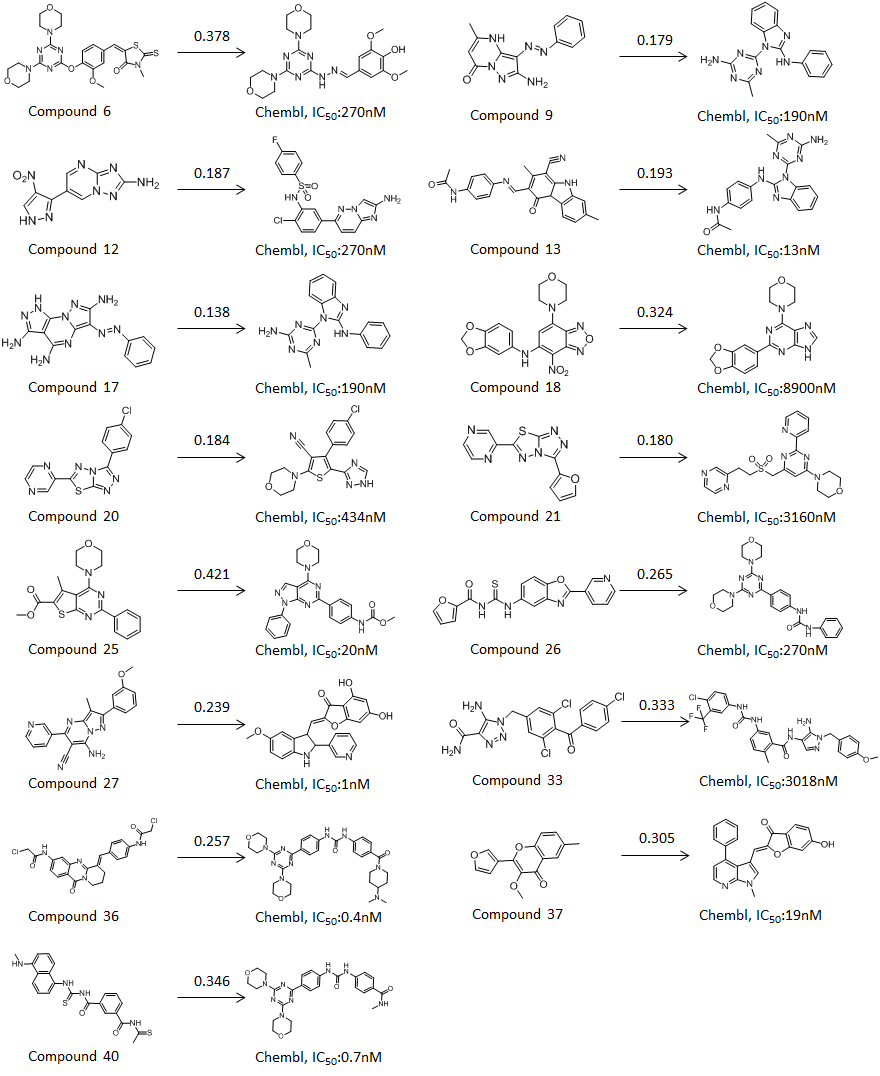


**Figure S3.** The closest compounds from ChEMBL to the confirmed mTOR inhibitors in the present study. All calculations were done in Discovery Studio 3.5.


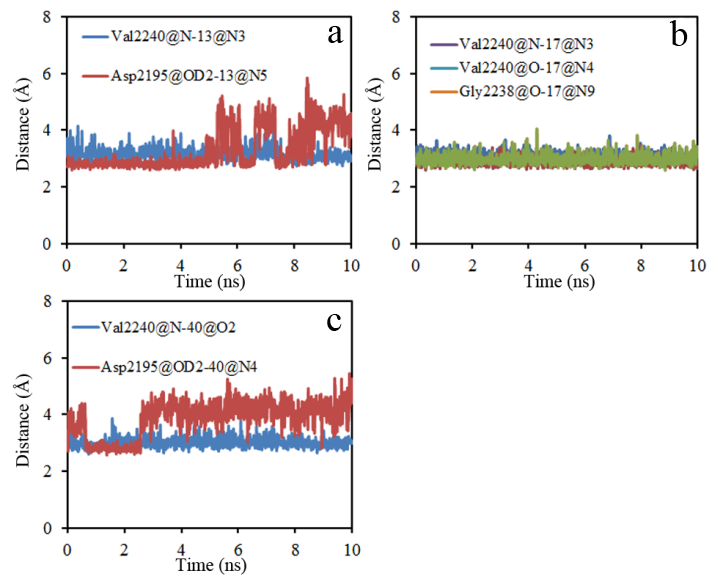


**Figure S4.** Time dependence of distance-based hydrogen bond analysis for **13**, **17** and **40** during 10 ns MD simulations.


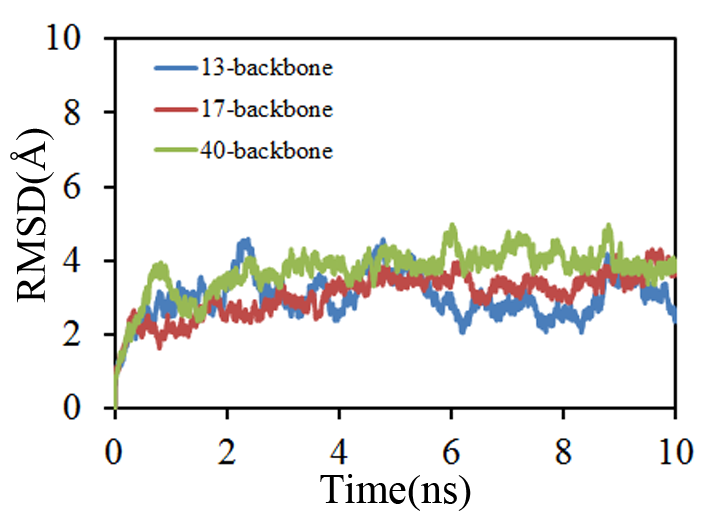


**Figure S5.** RMSD plot for the backbone atoms of **13**, **17** and **40**-mTOR complexes during 10 ns MD simulations after equilibration.


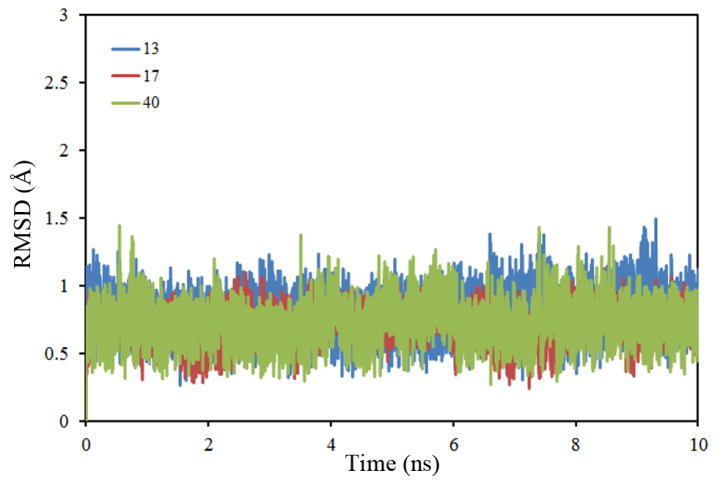


**Figure S6.** RMSD plot for the 13, 17 and 40 during 10 ns MD simulations after equilibration.

**Figure S7.** ^1^H NMR and MS data of selected compounds. All compounds tested in the present study are commercial available. Five compounds (**12**, **17,** **18**, **20**, **21**, and **26**) were randomly selected from active compounds for 1H NMR, and MS measurement. This sampling check method can be found in other virtual screening study (Yuk Yin Sham et al. *J. Med. Chem*, 2014, *57*, 1121–1126) and detailed results are given as follows:

^1^H-NMR spectrum of Compound **12** (Pyridine-d5, 400 MHz):


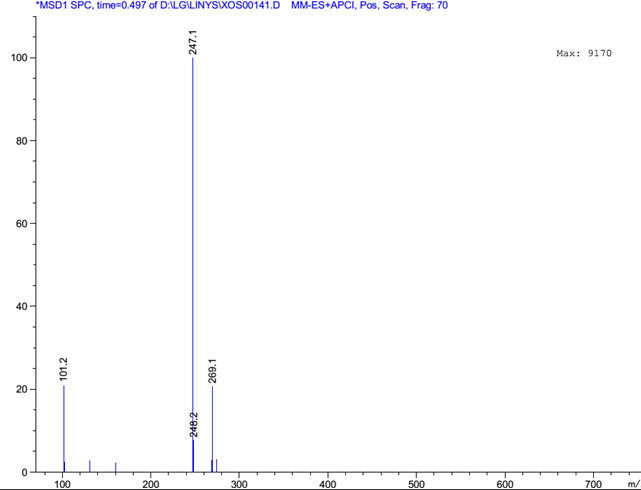


MS of Compound **12**

^1^H-NMR spectrum of Compound **18** (CDCl_3_, 400 MHz)


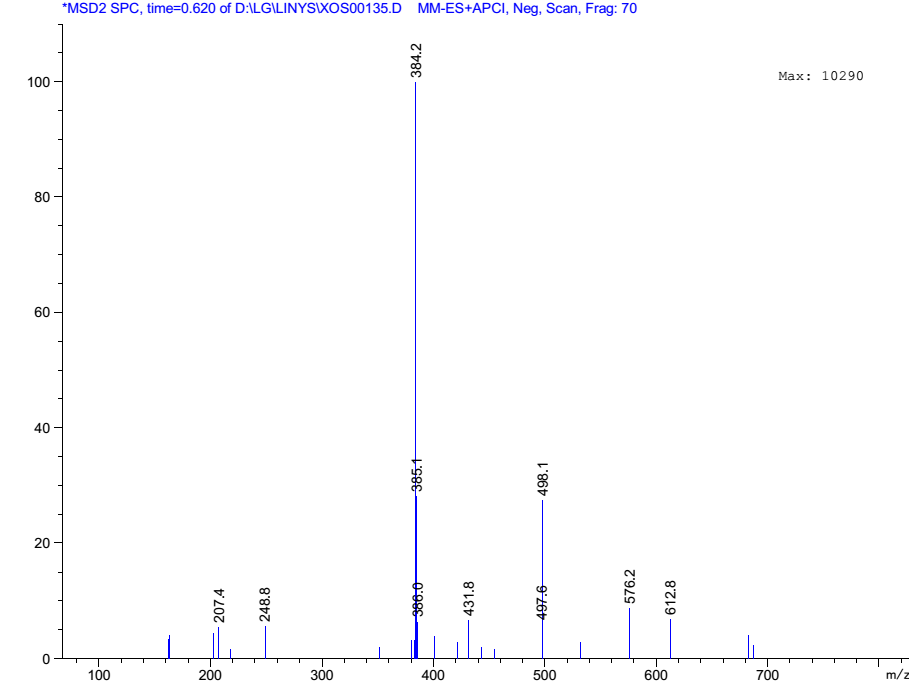


MS of Compound **18**

^1^H-NMR spectrum of Compound **20**(CDCl_3_, 400 MHz):


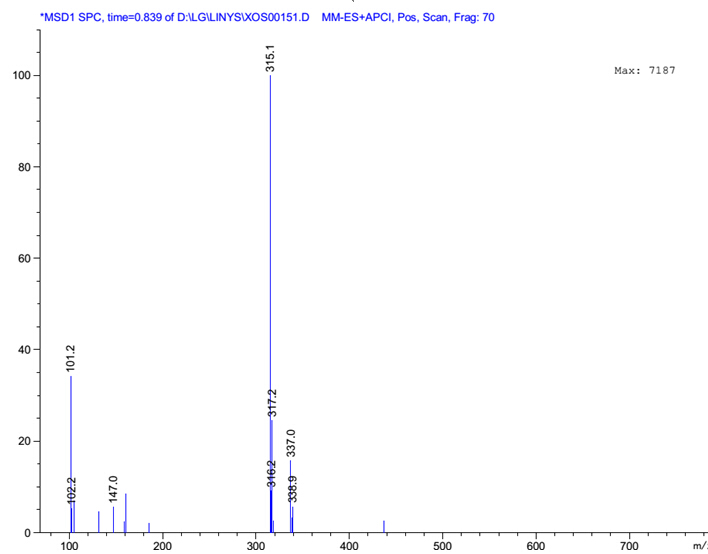


MS of Compound **20**

^1^H-NMR spectrum of Compound **21**(CDCl_3_, 400 MHz):


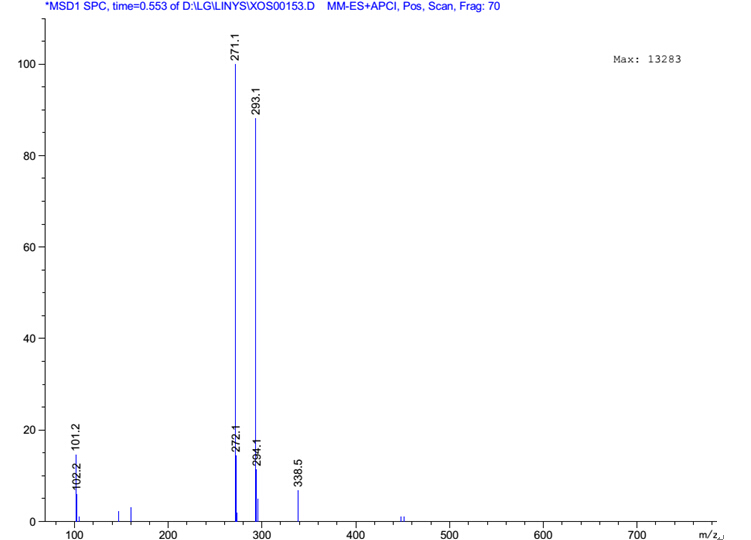


MS of Compound **21**

^1^H-NMR spectrum of Compound **26** (CDCl_3_, 400 MHz)


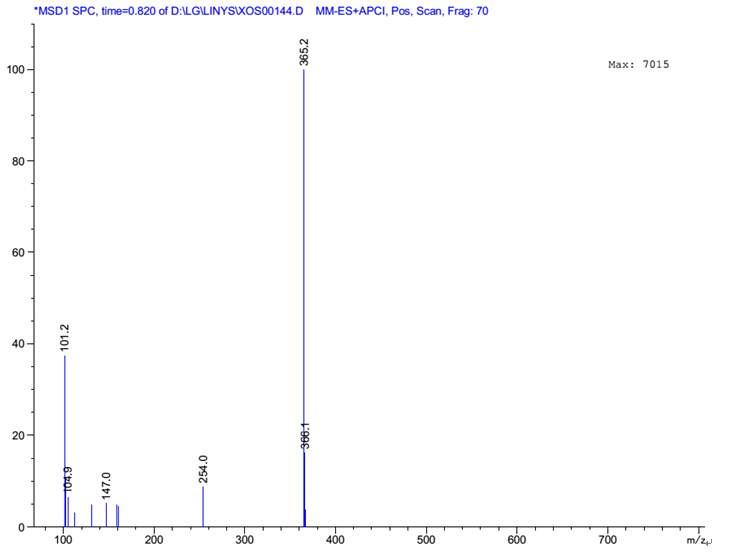


MS of Compound **26**

**Figure S8.** ^1^H NMR spectra of 17 supplied by SPECS Inc.

**
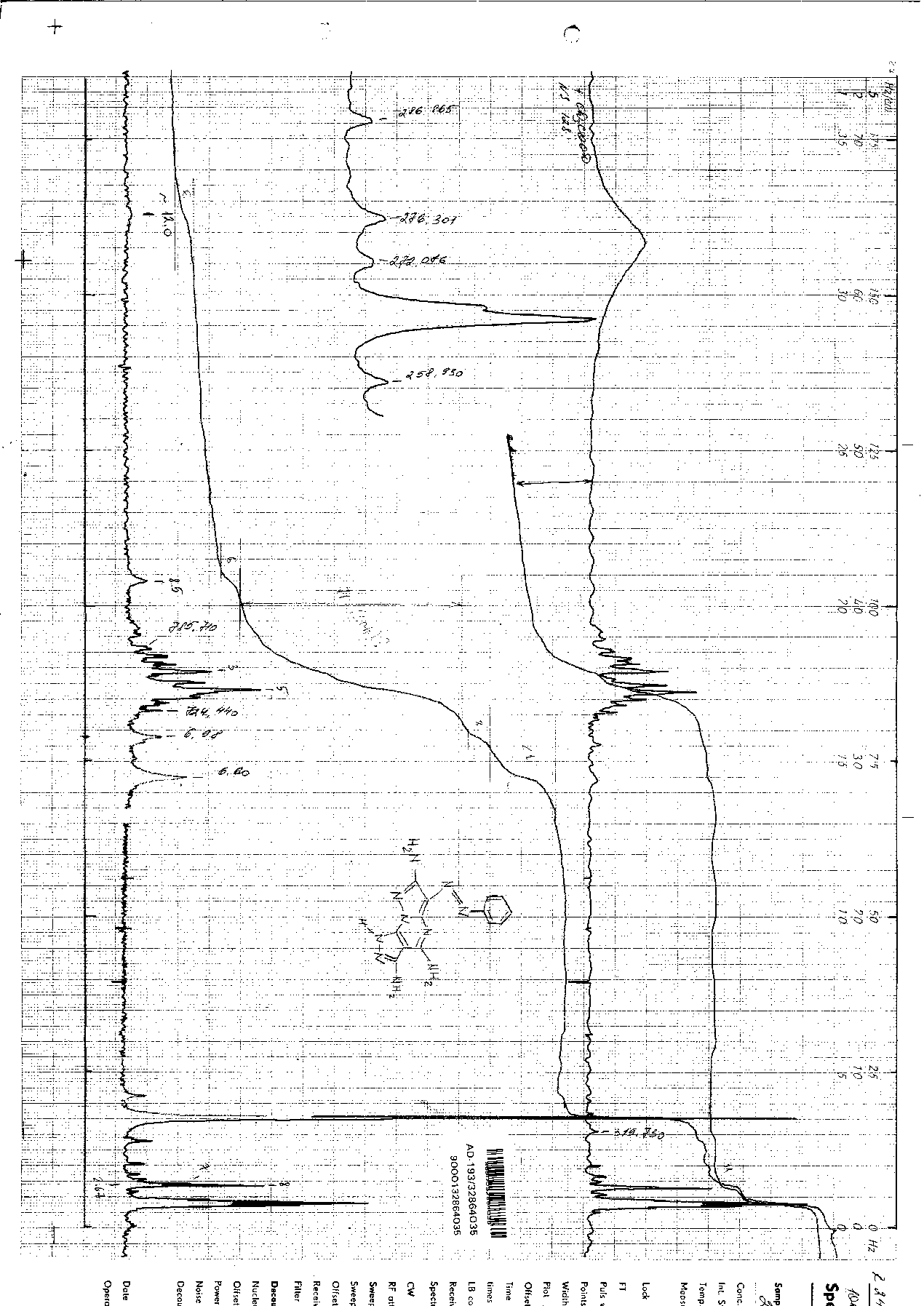
**

^1^H-NMR spectrum of Compound **17** (supplied by SPECS Inc.)

**Table S1.** HPLC analysis data of the 15 active compounds. The purity of hit compounds discussed in the text was determined by HPLC equipped with a XDB-C18 column (250×4.6 mm, 5 μm particle size) and a UV/VIS detector setting of λ=254 nm. All compounds were eluted with the two solvent systems (CH_3_OH as organic phase in Method I and CH_3_CN as organic phase in Method II).

| Compd. | Method I | Method II |
| --- | --- | --- |
| 6 | 0-20min, CH_3_OH/H_2_O=5:5 (v/v) retention time: 4.81 min relative purity: 97.99% | 0-20min, CH_3_CN/H_2_O=5:5 (v/v) retention time: 10.50 min relative purity: 97.85% |
| 9 | 0-20min, CH_3_OH/H_2_O=5:5 (v/v) retention time: 4.45min relative purity: 96.59% | 0-20min, CH_3_CN/H_2_O=5:5 (v/v) retention time: 6.49 min relative purity: 97.95% |
| 12 | 0-20min, CH_3_OH/H_2_O=2:8 (v/v) retention time: 3.84min relative purity: 96.04% | 0-20min, CH_3_CN/H_2_O=5:5 (v/v) retention time: 7.26min relative purity: 98.72% |
| 13 | 0-20min, CH_3_OH/H_2_O=6:4 (v/v) retention time: 6.38 min relative purity: 98.76% | 0-20min, CH_3_CN/H_2_O=5:5 (v/v) retention time: 11.77 min relative purity: 96.79% |
| 17 | 0-20min, CH_3_OH/H_2_O=6:4 (v/v) retention time: 10.42min relative purity: 96.20% | 0-20min, CH_3_CN/H_2_O=5:5 (v/v) retention time: 6.88 min relative purity: 99.52% |
| 18 | 0-20min, CH_3_OH/H_2_O=5:5 (v/v) retention time: 14.72 min relative purity: 99.06% | 0-20min, CH_3_CN/H_2_O=5:5 (v/v) retention time: 10.92 min relative purity: 98.06% |
| 20 | 0-20min, CH_3_OH/H_2_O=5.5:4.5 (v/v) retention time: 8.80 min relative purity: 96.99% | 0-20min, CH_3_CN/H_2_O=5:5 (v/v) retention time: 7.08 min relative purity: 99.08% |
| 21 | 0-20min, CH_3_OH/H_2_O=5.5:4.5 (v/v) retention time: 6.22 min relative purity: 98.00% | 0-20min, CH_3_CN/H_2_O=5:5 (v/v) retention time: 9.20 min relative purity: 97.47% |
| 25 | 0-20min, CH_3_OH/H_2_O=5:5 (v/v) retention time: 8.80 min relative purity: 95.64% | 0-20min, CH_3_CN/H_2_O=5:5 (v/v) retention time: 15.39 min relative purity: 98.35% |
| 26 | 0-20min, CH_3_OH/H_2_O=7:3 (v/v) retention time: 6.85min relative purity: 97.45% | 0-20min, CH_3_CN/H_2_O=5:5 (v/v) retention time: 6.75 min relative purity: 98.11% |
| 27 | 0-20min, CH_3_OH/H_2_O=6.5:3.5 (v/v) retention time: 9.61 min relative purity: 97.46% | 0-20min, CH_3_CN/H_2_O=4:6 (v/v) retention time: 9.24 min relative purity: 96.82% |
| 33 | 0-20min, CH_3_OH/H_2_O=5.5:4.5 (v/v) retention time: 6.31 min relative purity: 95.67% | 0-20min, CH_3_CN/H_2_O=5:5 (v/v) retention time: 6.40min relative purity: 95.99% |
| 36 | 0-20min, CH_3_OH/H_2_O=6:4 (v/v) retention time: 7.97 min relative purity: 97.88% | 0-20min, CH_3_CN/H_2_O=5:5 (v/v) retention time: 5.25min  relative purity: 95.56% |
| 37 | 0-20min, CH_3_OH/H_2_O=5:5 (v/v) retention time: 7.89 min relative purity: 95.48% | 0-20min, CH_3_CN/H_2_O=5:5 (v/v) retention time: 10.17min relative purity: 97.69% |
| 40 | 0-20min, CH_3_OH/H_2_O=5:5 (v/v) retention time: 1.48 min relative purity: 96.57% | 0-20min, CH_3_CN/H_2_O=5:5 (v/v) retention time: 4.51 min relative purity: 97.99% |

**Table S2. The corresponding number, docking score, source of database, ID and the ranks in Table 1 of the virtual screening hits after clustering for bioassays.**

| Compound NO | XP-Score | Source | ID | Rank |
| --- | --- | --- | --- | --- |
| Compound 1 | -8.259 | Specs | AL-281/41608991 | 22 |
| Compound 2 | -9.254 | Specs | AN-329/40261238 | 10 |
| Compound 3 | -9.073 | Specs | AG-670/41690963 | 13 |
| Compound 4 | -7.473 | Specs | AP-501/41557726 | 33 |
| Compound 5 | -11.131 | Specs | AG-690/11632011 | 2 |
| Compound 6*^a^* | -8.301 | Specs | AN-648/12988002 | 21 |
| Compound 7 | -7.177 | Specs | AG-670/15095010 | 37 |
| Compound 8 | -7.944 | Specs | AN-648/15101192 | 28 |
| Compound 9*^a^* | -8.753 | Specs | AI-031/31965023 | 15 |
| Compound 10 | -10.807 | Specs | AI-031/31965026 | 4 |
| Compound 11 | -7.471 | Specs | AG-205/32621058 | 33 |
| Compound 12*^a^* | -7.999 | Specs | AJ-326/34243011 | 27 |
| Compound 13*^a^* | -7.847 | Specs | AG-690/34447031 | 30 |
| Compound 14 | -10.633 | Specs | AO-476/43201417 | 6 |
| Compound 15 | -10.973 | Specs | AO-476/43250119 | 3 |
| Compound 16 | -7.498 | Specs | AP-501/43273811 | 32 |
| Compound 17*^a^* | -12.096 | Specs | AD-193/32864035 | 1 |
| Compound 18*^a^* | -8.691 | Specs | AG-227/40928271 | 16 |
| Compound 19 | -6.553 | Specs | AO-854/40805895 | 39 |
| Compound 20*^a^* | -9.496 | Specs | AO-365/43301198 | 9 |
| Compound 21*^a^* | -8.958 | Specs | AO-365/43300846 | 14 |
| Compound 22 | -8.508 | Specs | AO-476/40921662 | 19 |
| Compound 23 | -8.05 | Specs | AP-853/43368099 | 25 |
| Compound 24 | -8.686 | Specs | AP-970/43375155 | 16 |
| Compound 25*^a^* | -9.1 | Specs | AI-020/13900001 | 12 |
| Compound 26*^a^* | -10.185 | Specs | AP-970/40475435 | 7 |
| Compound 27*^a^* | -9.722 | Specs | AO-476/43415559 | 8 |
| Compound 28 | -10.655 | Specs | AO-476/15188007 | 5 |
| Compound 29 | -8.339 | Specs | AO-022/43453835 | 20 |
| Compound 30 | -8.159 | Specs | AT-047/43463789 | 24 |
| Compound 31 | -6.43 | GSMLT | sysu-23785S | 40 |
| Compound 32 | -8.041 | GSMLT | sysu-23739S | 26 |
| Compound 33*^a^* | -7.428 | GSMLT | sysu-20194S | 35 |
| Compound 34 | -8.598 | GSMLT | sysu-24206S | 18 |
| Compound 35 | -7.933 | GSMLT | sysu-24918s | 29 |
| Compound 36*^a^* | -9.216 | GSMLT | sysu-00760 | 11 |
| Compound 37*^a^* | -8.189 | GSMLT | sysu-01723 | 23 |
| Compound 38 | -7.679 | GSMLT | sysu-20435s | 31 |
| Compound 39 | -6.377 | GSMLT | sysu-00167 | 41 |
| Compound 40*^a^* | -7.41 | GSMLT | sysu-00174 | 36 |
| Compound 41 | -6.555 | GSMLT | sysu-20445s | 38 |

***^a^***Potent mTOR inhibitors determined by bioassays.

**Table S3.** Free energy analysis for the binding of **13**, **17**, **40** to mTOR.

| Energy terms | Binding free energy (kcal/mol) | | |
| --- | --- | --- | --- |
|  | 13 | 17 | 40 |
| ΔE_vdw_*^a^* | –-53.44 (2.80) | –45.51 (2.50) | –55.57 (2.98) |
| ΔE_ele_*^b^* | –24.14 (5.18) | –24.64 (4.11) | –22.49 (4.63) |
| ΔE_ele,solv_*^c^* | 41.00 (4.40) | 33.96 (2.65) | 38.35 (3.94) |
| ΔE_nonpol,solv_*^d^* | –6.42 (0.22) | –4.78 (0.17) | –6.55 (0.28) |
| ΔG_gas_*^e^* | –77.59 (5.27) | –70.15 (4.20) | –78.06 (5.11) |
| ΔG_solv_*^f^* | 34.58 (4.32) | 29.18 (2.60) | 31.80 (3.86) |
| ΔG_binding_*^g^* | –43.01 (2.83) | –40.98 (2.98) | –46.26 (2.98) |

*^a^*Nonbonded van der Waals. *^b^*Nonbonded electrostatics. *^c^*Polar component to solvation. *^d^*Nonpolar component to solvation. *^e^*Total gas phase energy. *^f^*Sum of nonpolar and polar contributions to solvation. *^g^*Final estimated binding free energy calculated from the terms above. Standard deviation values are shown in parentheses.

**Table S4. Percentages of enzymatic inhibitions by compound 17 (10 μM) on 24 structurally related kinases of mTOR.**

| Kinase | % Inhibition at 10 μM*^a^* |
| --- | --- |
| AKT1 (PKB alpha) | 22.69±9.34 |
| AKT2 (PKB beta) | 33.56±3.23 |
| AKT3 (PKB gamma) | 11.66±0.37 |
| AMPK A1/B1/G1 | 17.00±3.47 |
| AMPK A2/B1/G1 | 33.83±4.29 |
| CDK1/cyclin B | 3.09±2.31 |
| CDK2/cyclin A | -1.54±1.35 |
| DNA-PK | 61.91±6.43 |
| FRAP1 (mTOR) | 16.88±4.99 |
| IKBKB (IKK beta) | 11.73±1.62 |
| MARK1 (MARK) | -12.21±4.24 |
| PDK1 | 3.94±3.24 |
| PRKCA (PKC alpha) | 9.84±3.05 |
| PRKCB1 (PKC beta I) | 9.54±0.09 |
| PRKCB2 (PKC beta II) | 17.73±4.98 |
| PRKCD (PKC delta) | -10.48±9.55 |
| RPS6KB1 (p70S6K) | -1.25±1.53 |
| PI4KB (PI4K beta) | 31.84±1.18 |
| PIK3C2A (PI3K-C2 alpha) | 28.64±1.96 |
| PIK3C2B (PI3K-C2 beta) | 39.30 ±7.29 |
| PIK3C3 (hVPS34) | -6.75 ±8.78 |
| PIK3CA/PIK3R1 (p110 alpha/p85 alpha) | 56.43 ±4.16 |
| PIK3CD/PIK3R1 (p110 delta/p85 alpha) | 41.40 ±0.63 |
| PIK3CG (p110 gamma) | 40.88 ±1.19 |

*^a^*%Inhibition values are the mean ± SD of triplicate measurements at 10μM.

**MD simulations and Binding free energy analysis**

The docked structures of inhibitors (**13**, **17** and **40**) in complex with mTOR were used as the initial coordinates for MD simulations. The partial charges of three inhibitors and PP242 were computed using the HF/6-31 G* basis set from GAUSSIAN03[^1^](#_ENREF_1), and refined by RESP calculation using the antechamber module of the AMBER 12 package[^2^](#_ENREF_2). The crystal structure of mTOR-mLST8-pp242 complex consists of 1174 amino acids of mTOR (residues 1376–2549), 326 amino acids of target of rapamycin complex subunit LST8, and pp242 inhibitor.[^3^](#_ENREF_3) For MD simulations, LST8 and FAT subunit (residues 1376–1982) were deleted because they were far away from the ATP binding pocket of mTOR kinase domain. Moreover, a 55-residue unstructured segment (residues 2437–2491) was not added because it is not conserved and has not effect on mTOR activity[^3^](#_ENREF_3). mTOR kinase domain[^4^](#_ENREF_4) (ATP binding domain, ~512 residues) were obtained for MD simulations.

MD simulations consisted of energy minimization, heat phase, equilibration and production. During the first phase of the minimization, only water and ions atoms were relaxed for 2000 steps, holding all other atoms restrained with a harmonic restraint of 10 kcal mol^−1^Å^−2^. The second minimization phase is that the protein backbone atoms were restrained with a harmonic restraint of 5 kcal mol^−1^Å^−2^ and hydrogen atoms, water molecules and ions were relaxed with 2000 steps. In the last minimization phase, all atoms were freely minimized with 2000 steps. In each minimization, a combined steepest descent and conjugate gradient minimization steps was equal. Subsequently, the system was linearly heated to 310 K with 50 ps NVT ensemble using a langevin thermostat with harmonic restraints of 10 kcal mol^−1^Å^−2^ on the backbone atoms. Then, a further 100 ps run at 310 K was conducted in NPT ensemble with 5 kcal mol^−1^Å^−2^, and pressure controlled using a Berendsen barostat[^5^](#_ENREF_5) with a coupling constant of 1 ps and a target pressure of 1 atm. The system was again equilibrated to simulation for 100 ps with a harmonic restraint of 0.5 kcal mol^−1^Å^−2^ for backbone atoms. Finally, a production simulation run for 10 ns was performed at 310 K. All hydrogen atoms were constrained using the SHAKE algorithm[^6^](#_ENREF_6) and the time step was set at 2 fs. Long-range electrostatic interactions were included on every step using the Particle Mesh Ewald algorithm[^7^](#_ENREF_7). Coordinate trajectories were recorded every 1 ps.

For each system, binding free energy calculations was performed on 500 snapshot structures extracted at 4 ps intervals over the last 2 ns stable MD trajectory via the molecular mechanics generalized Born surface area (MM-GBSA) method.

In the MM-GBSA approach an interaction free energy is defined as

△*G*_binding_ =*G*_complex_–[*G*_protein_ + *G*_ligand_] (1)

Where *G*_complex_, *G*_protein_, and *G*_ligand_ are the free energies of the complex, protein and the ligand, respectively. Each free energy term in eq 1 was computed as sum of the absolute free energy in the gas phase (*E*_gas_), the solvation free energy (*G*_solvation_), and the entropy term (*TS*), using eq 2:

*G* =*E*_gas_+ *G*_solvation_- *TS* (2)

*E*_gas_ was expressed as the sum of changes in the van der Waals energy (*E*_vdw_), electrostatic energy (*E*_ele_), and the internal energies (*E*_int_) in the gas phase (eq 3). *E*_int_ is the energy associated with vibration of covalent bonds and bond angels, rotation of single bond torsional angels (eq 4)

*E*_gas_ = *E*_int_+*E*_vdw_+ *E*_ele_ (3)

*E*_int_ =*E*_bond_+*G*_angel_ +*E*_torsion_ (4)

The solvation free energy, *G*_solvation_, is approximated as the sum of the polar contribution (*G*_GB_) and nonpolar contribution (*G*_nonpolar_) using continuum solvent methods:

*G*_solvation_= *G*_GB_ + *G*_nonpolar_ (5)

*G*_nonpolar_= γ×SASA + *b* (6)

The polar contribution (*G*_GB_) to the solvation energy was calculated using GB model implemented in AMBER 12. The grid size used is 0.5 Å. The dielectric constant was set to 1 for interior solute and 80 for exterior water. The nonpolar contributions (*G*_nonpolar_) were estimated using eq6, where SASA is the solvent-accessible surface area that was estimated using the linear combination of pairwise overlaps (LCPO)[^10^](#_ENREF_10); the probe radius of 1.4 Å, γ= 0.0072 kcal·mol^-1^·Å^-2^, and *b*=0 kcal/mol (eq 6).

The calculation of the entropic contribution is computationally expensive and omitted in our study because it requires extremely well minimized structures for a normal-mode analysis or large numbers of snapshots for a quasi-harmonic analysis[^11^](#_ENREF_11). The binding free energy decomposition was performed on a per-residue basis using the MM-GBSA method[^12-14^](#_ENREF_12). This decomposition was carried out only for molecular mechanics and solvation energies but not for entropies.

**References**

1. M. J. Frisch G. W. T., H. B. S., G. E. Scuseria, M. A. Robb. Gaussian 03, Revision E.01, Gaussian, Inc, Pittsburgh PA. 2004.

2. Salomon-Ferrer, R., Case, D. A. & Walker, R. C. An overview of the amber biomolecular simulation package. *Wiley Interdiscip. Rev. Comput. Mol. Sci.* **3**, 198-210 (2013).

3. Yang, H. J. *et al.* mTOR kinase structure, mechanism and regulation. *Nature* **497**, 217-223 (2013).

4. Khanfar, M. A., AbuKhader, M. M., Alqtaishat, S. & Taha, M. O. Pharmacophore modeling, homology modeling, and in silico screening reveal mammalian target of rapamycin inhibitory activities for sotalol, glyburide, metipranolol, sulfamethizole, glipizide, and pioglitazone. *J. Mol. Graph. Model.* **42**, 39-49 (2013).

5. Berendsen, H. J. C., Postma, J. P.M., Vangunsteren, W. F., Dinola, A. & Haak, J. R. Molecular-dynamics with coupling to an external bath. *J. Chem. Phys.* **81**, 3684-3690 (1984).

6. S. Miyamoto, P. A. K. Settle: an analytical version of the SHAKE and RATTLE algorithm for rigid water models. *J. Comput. Chem.* **13**, 8952-8962 (1992).

7. Norberto de Souza, O. & Ornstein, R. L. Effect of warmup protocol and sampling time on convergence of molecular dynamics simulations of a DNA dodecamer using AMBER 4.1 and particle-mesh Ewald method. *J. Biomol. Struct. Dyn.* **14**, 607-611 (1997).

8. Kollman, P. A. *et al.* Calculating structures and free energies of complex molecules: combining molecular mechanics and continuum models. *Acc. Chem. Res.* **33**, 889-897 (2000).

9. Swanson, J. M. J., Henchman, R. H. & McCammon, J. A. Revisiting free energy calculations: A theoretical connection to MM/PBSA and direct calculation of the association free energy. *Biophys. J.* **86**, 67-74 (2004).

10. Weiser, J., Shenkin, P. S. & Still, W. C. Approximate atomic surfaces from linear combinations of pairwise overlaps (LCPO). *J. Comput. Chem.* **20**, 217-230 (1999).

11. Perez, M. A. S., Sousa, S. F., Oliveira, E. F. T., Fernandes, P. A. & Ramos, M. J. Detection of farnesyltransferase interface hot spots through computational alanine scanning mutagenesis. *J. Phys. Chem. B.* **115**, 15339-15354 (2011).

12. Tsui, V. & Case, D. A. Theory and applications of the generalized Born solvation model in macromolecular Simulations. *Biopolymers* **56**, 275-291 (2000).

13. Rastelli, G., Del Rio, A., Degliesposti, G. & Sgobba, M. Fast and accurate predictions of binding free energies using MM-PBSA and MM-GBSA. *J. Comput. Chem.* **31**, 797-810 (2010).

14. Zoete, V., Irving, M. B. & Michielin, O. MM-GBSA binding free energy decomposition and T cell receptor engineering. *J. Mol. Recognit.* **23**, 142-152 (2010).
